# Together with 30 years of Symposia on Chrysomelidae! Memories and personal reflections on what we know more about leaf beetles

**DOI:** 10.3897/zookeys.547.7181

**Published:** 2015-12-17

**Authors:** Pierre Jolivet

**Affiliations:** 1 67 boulevard Soult, F-76012 Paris, France

## Introduction

Certainly, Carabidae, Curculionidae and Chrysomelidae are the beetle families that are most studied and the most inspiring for scientific papers. Those three families are also among the most numerous and present the most colorful beetles. Publications go from simple articles in the past to sophisticated papers using cladistics, molecular biology and statistics, in pure research or, for leaf-beetles or weevils, in agriculture. Thousands of papers are published each year on Chrysomelidae. Probably the actual described number of Chrysomelidae, estimated last century as 35.000 species, reaches 45.000 and there probably exist 55.000 to 60.000 species around the world. Canopy species are among the least known, true also for minute species living in litter or mosses

**Figure 1.**
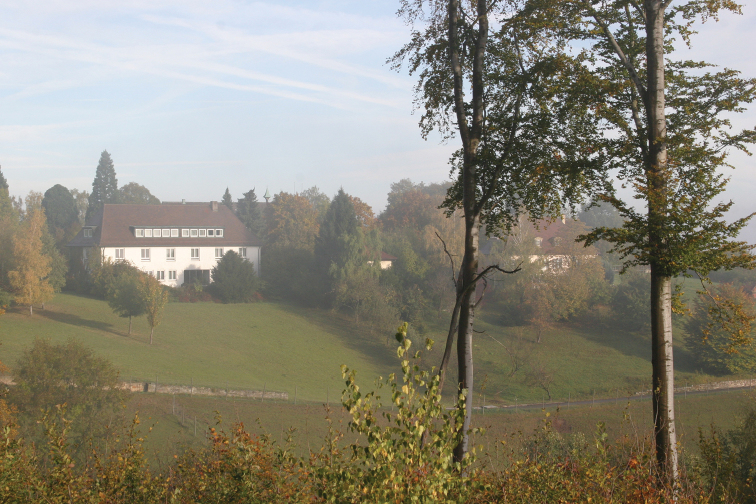
View of the venue of the meetings of the German-speaking coleopterists, Landgut Burg vicinity of Beutelsbach, 24.10.2009, southwest Germany (near Stuttgart, photo: M. Schmitt).

**Figure 2.**
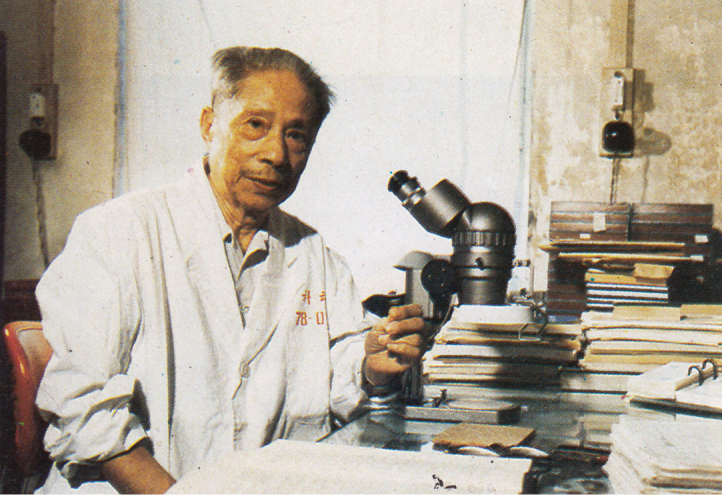
Prof. Shi-xiang Chen (5.11.1905 – 25.1.1988), from a booklet of the Chinese Academy of Sciences 1992.

Coleoptera can easily exceed 1 to 2 million species and, in the past (in the Mesozoic, but mostly in Cenozoic), they must have been much more numerous. Only Curculionidae and perhaps Staphylinidae can surpass the number of Leaf-Beetles. Curculionidae are present everywhere, even in the sub-Antarctic islands and in Greenland, where Chrysomelidae are missing, even if present there during the Pliocene. Still many species of weevils remain to be described, among the endogeous, myrmecophilous, floricolous species. Symposia on Leaf Beetles, originally organized every four years, now perhaps every two years, together with International and European Congresses of Entomology, or independently, generally are published later in books, which tend actually and only very recently to be published electronically. Many international publishers were responsible for those books and we are indebted to many specialists and co-editors. There were also regular annual meetings of chrysomelid specialists in the United States, correlated with the Entomological Society of America meetings, grouping often part of the specialists from the previous symposia, the next one probably coinciding with the International Congress of Entomology in 2016. Annual meetings of chrysomelid workers were also held in Japan each year. One chrysomelid symposium was organized in Patiala, India, with 29 papers in March, 1989. Sporadic chrysomelid symposia are also held with French and Belgian workers in Paris or elsewhere, in Costa Rica, with Wills Flowers, as in 1995, etc. Regular meetings take place each year in Germany together with the meetings (58 actually) of German-speaking coleopterists. Many European chrysomelidologists attend it also. Those German meetings actually are held in Beutelsbach (Fig. 1), on a hill, in a charming country inn, with a big meeting room and all video facilities. There were also meetings on Chrysomelidae in Pretoria, Republic of South Africa, connected with the local Entomological Society, in Milano, Italy, in Uberlandia, in Brazil, in 2005, etc. The Academia Sinica in China has been and is an active centre of leaf beetle research under Shi-xiang Chen (Fig. 2) and his successors. The death of Chen in 1988 was a big loss for the chrysomelidologists, but new generations have taken up the torch.

So, progress of our knowledge on the Chrysomelidae, on taxonomy, distribution, physiology, biology has been relatively fast the last 30 years. A Newsletter, devoted to leaf beetles, *Chrysomela*, was founded in 1979 by Terry Seeno and Eric Smith. It is still alive, now entirely in colour, with a new editor, Caroline Chaboo, and that has been also a stimulant for all chrysomelid lovers.

The enormous Georg Frey Collection of beetles (originally housed on the Frey estate in Tutzing) is now in Basle Museum, Switzerland. The Frey Chrysomelidae were initiated in Munich by Jan Bechyné, and most of those beetles are authoritatively identified, but unfortunately a general collection, a former UN dream, has never been made assembling all world insect types. Those types are mainly in the primary museums in London, Paris, Berlin, Munich, Moscow, Basle, Washington, Honolulu, Canberra, Beijing, Brussels, Tervueren, Tokyo, Pretoria, Maracay, Sao Paulo, Rio de Janeiro, and several other big or smaller collections. Due to possible damage in the mail, saving collections staff time and to hastened receipt, museums now try to send excellent digital photographs instead of the specimens themselves. On the spot, examination, remains always possible. Jesús Gómez-Zurita for instance visited the National Museum of Prague (with Achard collections) to see the Bechyné *Timarcha* types in 1997. Which resulted many excellent papers on the genus, its classification, and many molecular biology studies.

## Symposia history

**Figure 3.**
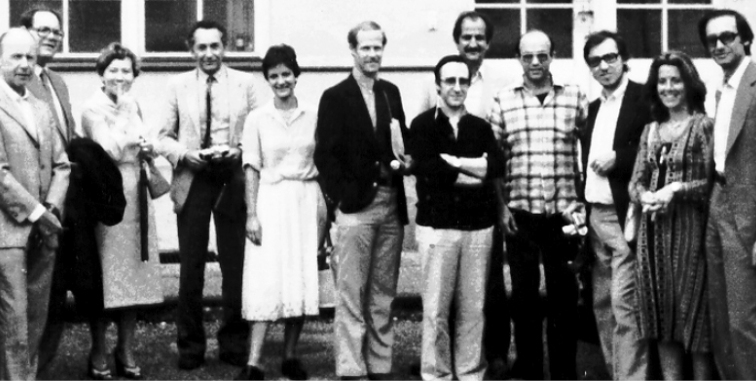
"The First Chrysomela Photo", showing the participants of the "First Internanional Alticinae Workers’ Symposium", Munich, August 1980: Walter Steinhausen, Manfred Doeberl, Bohumila Bechyné, Gerhard Scherer, Carmen Segarra, David Furth, Carlo Leonardi, Terry Seeno, Mauro Daccordi, Serge Doguet, Carmelèn Petitpierre, Eduard Petitpierre. From Chrysomela Newsletter 38/39 (2000, photo probably by Eric Smith).

The first symposium on Chrysomelidae Alticinae (Scherer 1982), was held in Munich (Fig. 3), mid- August 1980. It brought together 12 specialists, and curiously this symposium has not been counted as Symposium on Chrysomelidae no. 1. That could be, if I can say so, Symposium no. 0. It brought together some of our leaders as Bohumila Bechyné, representing her husband Jan, who had died on 9^th^ of March, 1973. Jan was a big describer of leaf beetles, a *Timarcha* lover, and, in some way, a pioneer in recent chrysomelid taxonomy. Most of those participants are still active actually. Some, as Gerhard Scherer and the Bechynés, have passed away. Good old times when in 1980 started *Chrysomela* story, with a general review of the taxonomy of the alticines. *Chrysomela* newsletter (actually # 54) started with 74 entomologists, and, despite more than twenty deaths (Enrique Balcells, Michio Chûjô, the Bechynés, Roy A. Crowson, J. Gordon Edwards, Dieter Erber, Nicole Berti, Michel Bergeal, Serge Iablokoff-Khnzorian, Shinzaku Kimoto, René Paulian, Sandro Ruffo, Igor Lopatin, Gerhard Scherer, Ray Smith, Niilo Virkki, John Wilcox, Krishna Kumar Verma, Yu Peiyu, Laurent LeSage and perhaps few others) the subscriptions actually reach 276!

**Figure 4.**
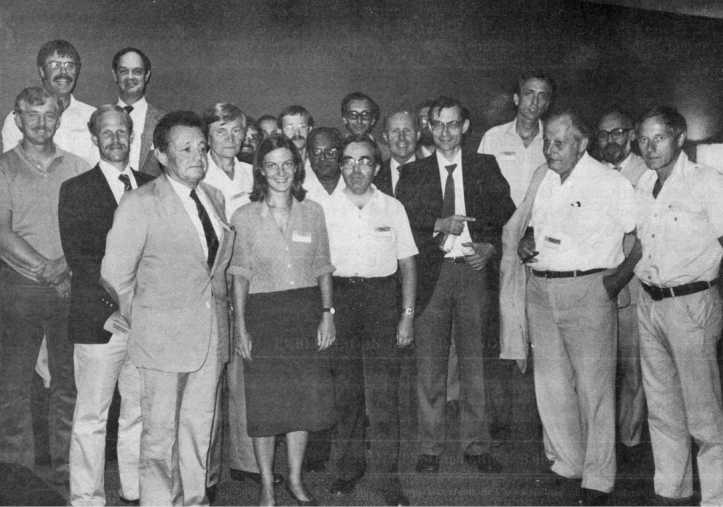
Group photo of the First International Symposium on the Chrysomelidae, Hamburg 1984 (from left): Arthur J. Gilbert, Ingolf Askevold, David Furth, Eric Smith, Pierre Jolivet, Niilo Virkki, Michael Schmitt, Hans Kroker (hidden), Carmen Segarra, Klaus Hemmann, Krishna K. Verma, Eduard Petitpierre, Hans Silfverberg, Walter Steinhausen, J. Watt, Horst Kippenberg, Felix Breden, Gustav Adolf Lohse, Brian Selman, Dieter Erber. From Chrysomela Newsletter 12 (1984).

The so-called First International Symposium on the Chrysomelidae (Fig. 4) was organized, in August 20–25, 1984, by David Furth, in Hamburg, Germany, together with the 17th International Congress of Entomology. A paper on the phylogeny of Chrysomelidae by Sicien H. Chen was presented. It’s really funny how the classification of the subfamilies evolved since Jacoby established in his time the first solid and long-time valid classification after Chapuis. Interesting papers on classification of Donaciinae by Ingolf Askevold, of Alticinae by David Furth, of Criocerinae by Michael Schmitt, of all the subfamilies by Kunio Suzuki, as well as on change of colour after death among Paropsini by Brian Selman were presented together with 22 other interesting papers. The symposium was published (Furth and Seeno, editors) in 1985. As part of most of these Congress Symposia, there were organized field excursions, with the authorization to collect some local insects. In 1988, the first volume on the Biology of the Chrysomelidae was published (JPH 1988).

**Figures 5, 6.**
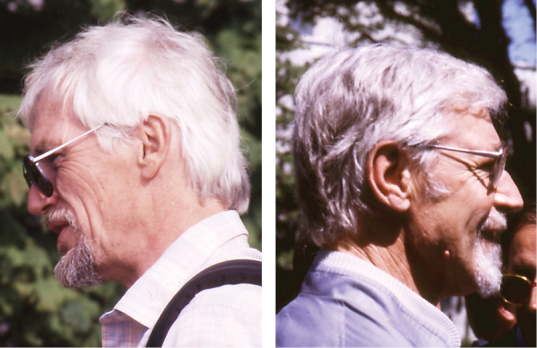
**5** (left) Al Samuelson, Vancouver 10.07.1988 (photo: M. Schmitt) **6** (right). G. Kuschel, Vancouver 9.07.1988 (photo: M. Schmitt).

It is with the Second International Symposium on the Chrysomelidae (8–9 July 1988, Furth and Seeno 1988), in Vancouver, Canada, that cycloalexy was borne. That was a joint idea of Joao Vasconcellos-Neto and myself, and *Coelomera*, *Chelymorpha* and *Platyphora* behaviour in Brazil gave us the idea. This symposium with 23 participants was held during two days, and Brian Farrell talked about leaf beetle community structure in Amazonian forest and Al Samuelson (Fig. 5) about pollen feeding in Alticinae. Some common interactions were done with the curculionidologists, namely with Willy Kuschel (Fig. 6) about soft wing structure. Dan Janzen made a brief appearance between two planes. He gave a bright talk on biodiversity, on his reforestation project in Costa Rica, asked for money and complained about the cost of our Conference: 6 million dollars. He was wearing leather boots, a hat on his head and was dressed as a true “Indiana Jones”. According to Ross Arnett, this was typical attire. I stopped him on his way to the airport, and the answer to my question: what about myrmecophytes? was “I will not do anything on ants before the coming 25 years!”

**Figure 7.**
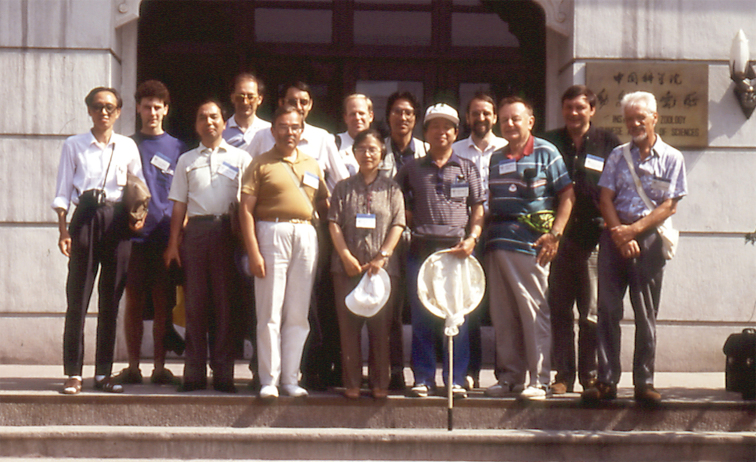
Group photo of the Third International Symposium on the Chrysomelidae, Beijing, 4 July, 1992, in front of the Academia Sinica (from left): Shu-yong Wang, Peter Verdyck, Shizuo Fujiyama, Eduard Petitpierre, Hans Silfverberg, Lech Borowiec, David Furth, Pei-yu Yu, Kunio Suzuki, Ting Hsiao, Michael Schmitt, Pierre Jolivet, Jacques Pasteels, Al Samuelson (photo: M. Schmitt).

The Third International Symposium on the Chrysomelidae (Furth 1994) was held in Beijing (Fig. 7), in July 1992, organized by David Furth and Yu Peiyu, in a big convention centre. It was very enjoyable with an all-day collecting excursion near the Great Wall, where we captured interesting Chrysomelinae, and a local Beijing field trip to Yuan Park where we collected beetles, and had a lot of scientific communications. At the end, Dr. Yu Peiyu organized an unforgettable classical roasted duck dinner. Petitpierre exposed his ideas on phylogenetic relationships among Chrysomelidae subfamilies. At that time, the Chrysomelidae remained all in the same family. No Megalopodidae, no Orsodacnidae, no Spilopyrinae, but still survived then the Megascelidinae.

**Figure 8.**
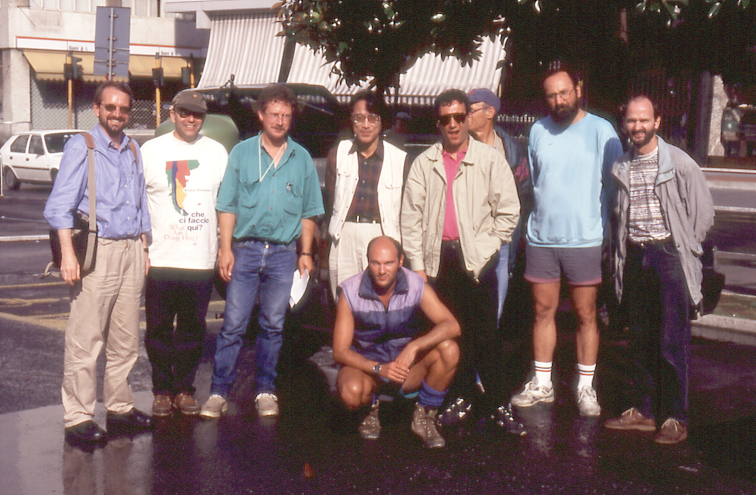
Part of the excursion group, after the Fourth International Symposium on the Chrysomelidae, Florence (Italy), 1 September, 1996 (from left): Michael Schmitt, Mauro Daccordi, Ron Beenen, Kunio Suzuki, Alessandro Bramanti, Maurizio Biondi, Roberto Bramanti, Károly Vig, Jörg Perner (photo: M. Schmitt).

The Fourth International Symposium on the Chrysomelidae was held as part of the XX International Congress of Entomology (ICE) in Florence, Italy from 25-31 August 1996. The symposium was organized by David Furth and Maurizio Biondi, and many entomologists from the whole planet attended. That was still at the end of the 30 "glorious years", and people had more money and still high level of security. The symposium was published in Italy, in Torino, by M. Biondi, M. Daccordi and D. G. Furth (editors) in 1998. Many formal presentations were done on various topics. Michael Schmitt showed photos from the previous two symposia. The excursion was to the Apuanian Mountains (Fig. 8), to find a new *Timarcha* from Mauro Daccordi. Some participants were lost and part of us did not see the famous *Timarcha apuana*. Michael Cox was there with his wife. Timarchologists and amateurs were all in search of *Timarcha*. Only one was captured! I like Italian cuisine and I enjoyed every evening spaghetti meat sauce (pasta Bolognese). The lunch with sandwiches on the spot was, on the contrary, not very attractive.

**Figure 9.**
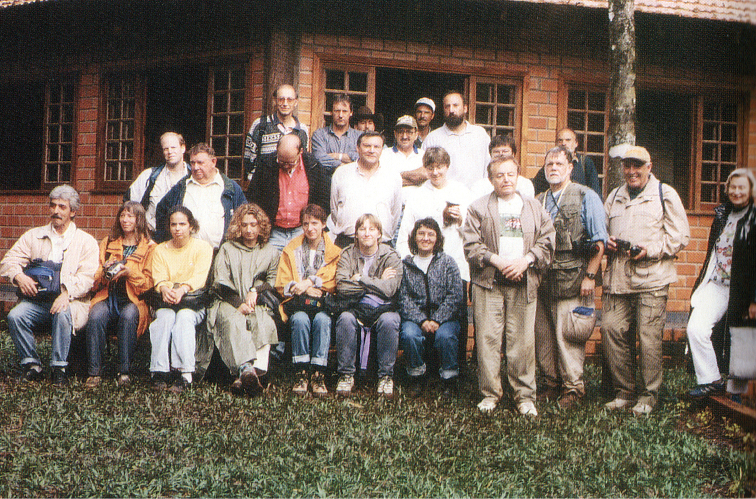
Fifth International Symposium on the Chrysomelidae, the participants of the after-congress excursion, Iguassu (Brazil), photo from Jolivet (2006).

The Fifth International Symposium on Chrysomelidae from August 25-27, 2000, was held in Iguassu Falls, Brazil (Fig. 9), a beautiful spot which I had visited already twice before. The symposium was organized by David Furth and Joao Vasconcellos-Neto. Many interesting papers were presented. The field trip was done in the Cabeza de Cachorro reserve, in the state of Paraná. During the trip, news arrived that it was suddenly forbidden to collect insects. The Proceedings of this Symposium were edited by David Furth as a book with Pensoft *Special Topics in Leaf Beetle Biology*, printed in 2003.

**Figure 10.**
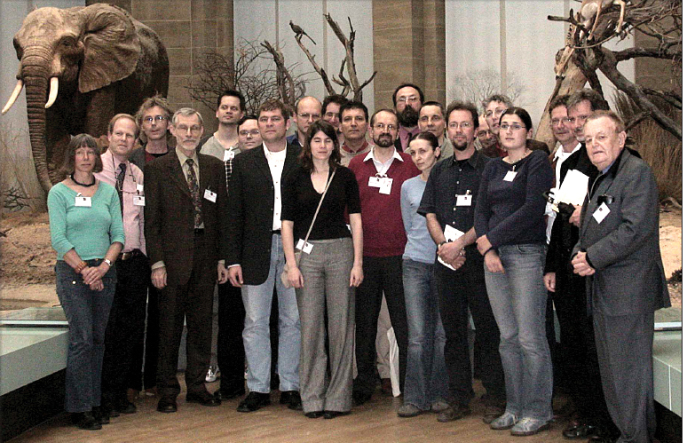
Sixth International Symposium on the Chrysomelidae, Bonn (Germany), 7 May, 2004, group photo in the great hall of the Zoologisches Forschungsmuseum Alexander Koenig, Bonn (from): Eva Sprecher-Übersax, David Furth, Jaap Winkelman, Horst Kippenberg, Wolfram Freund, Helmut Bolz, Jürgen Gross, Thomas Wagner, Susanne Düngelhoef, Lasse Hubweber, Maurizio Biondi, Michael Schmitt, Károly Vig, Jolanta Swietojanska, Lech Borowiec, Matthias Schöller, Mauro Daccordi, Elisabeth Geiser, Gudrun Fuss, Ron Beenen, Fredric Vencl, Pierre Jolivet (photo: M. Jolivet).

**Figure 11.**
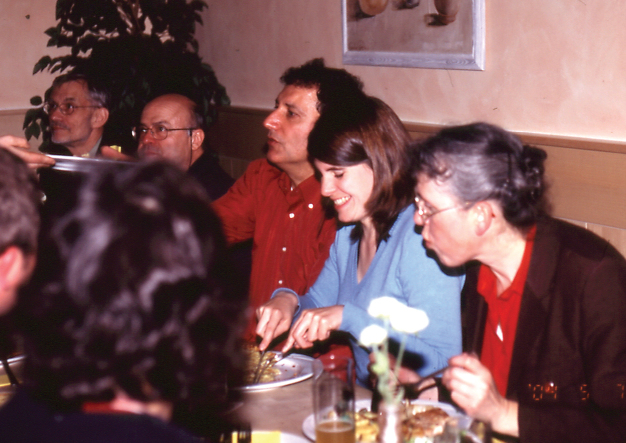
In the brew-pub, 7 May, 2004: Horst Kippenberg, Mauro Daccordi, Maurizio Biondi, Susanne Düngelhoef, Elisabeth Geiser (photo: M. Schmitt).

The Sixth International Symposium on the Chrysomelidae (Fig. 10) was held at the Museum Alexander Koenig, Bonn, Germany on 7th of May, 2004, with 22 participants from 10 countries. The 2004 ICE in Brisbane would not accept a symposium on only the Chrysomelidae. This meeting replaced the failure of the Prague Conference. It was organized by Michael Schmitt and connected with the Symposium on Tropical Biology. We had a joint dinner (Fig. 11) at a brew-pub, and we saw Beethoven house, the next day. As usual, interesting talks were held in a room near the former parliament of Germany, close to a historical giraffe. On Saturday, 8^th^ of May, an excursion was made near Koblenz for collecting, but in the rain. Proceedings of the Sixth International Symposium were published by Michael Schmitt as a special issue of Bonner zoologische Beiträge in 2006 (vol. 54/-4).

Some kind of Symposium (6a) or a virtual Symposium on Chrysomelidae, without any number, was held in Brisbane, Australia with the International Congress of Entomology on 16-21 August 2004 and attended by Chris Reid who wrote a review for *Chrysomela* (Reid 2004). John Lawrence was present and co-organizer. 26 papers on Chrysomelidae were presented. A formal dinner, rather expensive, though very spartan (in my table they brought food for 5 people when we were 6), closed the meetings. In 2004, Caroline Chaboo took over the newsletter *Chrysomela*, formerly edited and published by Terry Seeno.

**Figure 12.**
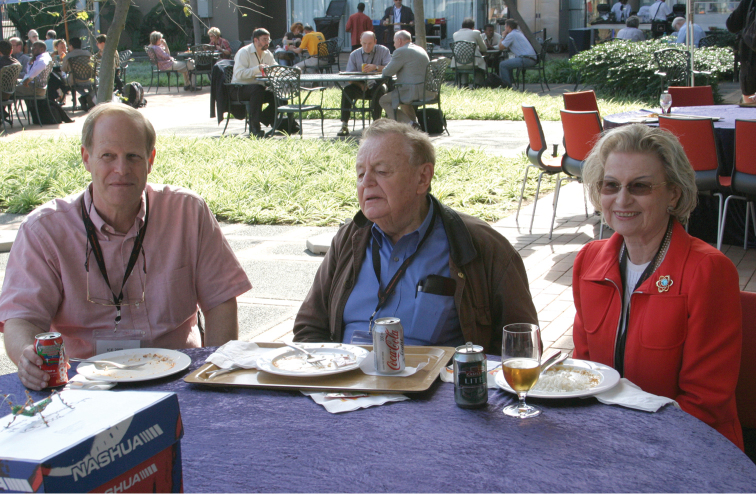
During the 23^rd^ International Congress of Entomology, Durban (South Africa), 9 July, 2008: David Furth, Pierre Jolivet, Madeleine (Mayon) Jolivet (photo: M. Schmitt).

**Figure 13.**
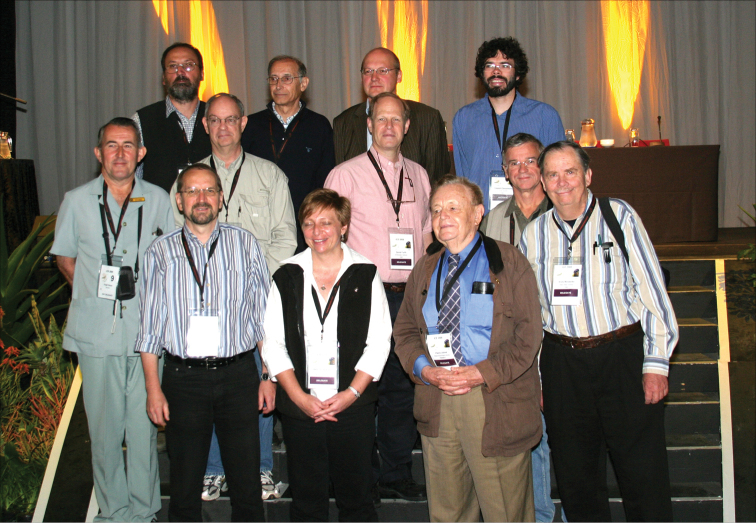
In the Great Hall of the Durban International Convention Centre, 9 July, 2008 (from left): Hugh D.C. Heron, Károly Vig, Michael Schmitt, Eric Smith, Eduard Petitpierre, Elizabeth Grobbelaar, Thomas Wagner, David Furth, Pierre Jolivet, Gaylord Desurmont, Gunter Maywald, Andrew Moldenke (photo: M. Schmitt).

The real Seventh International Symposium on the Chrysomelidae was held on July 9 in Durban, South Africa in connection with the 23^rd^ International Congress of Entomology (July 6-12, 2008). We had a big hall of more than 2500 seats for 20 people. It was co-organized by Michael Schmitt and Beth Grobbelaar. We talked about many aspects of leaf beetle biology, and I spoke on New Caledonia where I had made 6 visits for collecting Chrysomelidae (Fig. 12). Andrew Moldenke, the Clytrine specialist, was present, but did not talk about leaf beetles (Fig. 13).

It is the volume 2 of Research on Chrysomelidae (Brill publisher, 2009) which contains the proceedings of 7th International Symposium on Chrysomelidae.

At this period started the new series of books Research on Chrysomelidae co-edited by P. Jolivet, J. Santiago-Blay and M. Schmitt with Brill. Later on Pensoft took over, and actually four volumes have been printed, the present one is the fifth, a sixth is in preparation.

**Figure 14.**
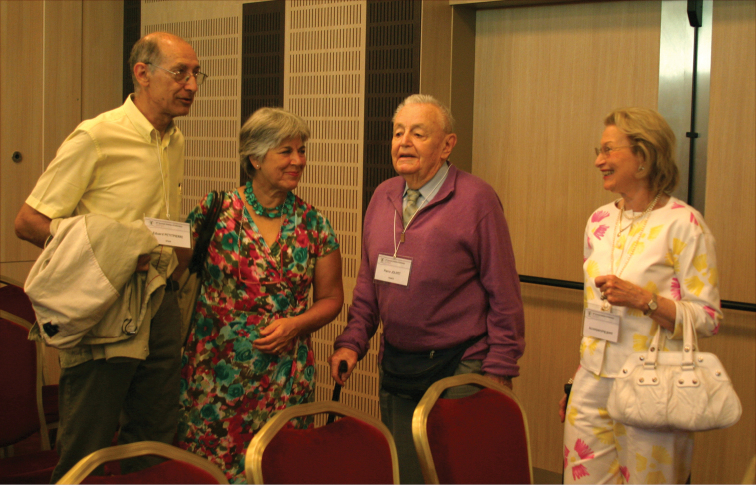
The First European Symposium on the Chrysomelidae, Budapest (Hungary), 23 August, 2010: Eduard Petitpierre, Carmelèn Petitpierre, Pierre Jolivet, Madeleine Jolivet (photo: M. Schmitt).

**Figure 15.**
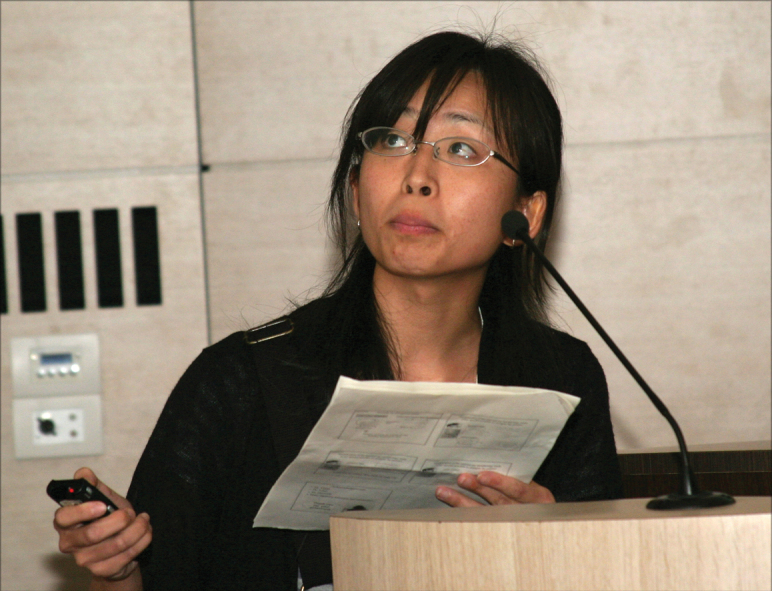
The First European Symposium on the Chrysomelidae, Budapest (Hungary), 23 August, 2010: Yoko Matsumura (photo: M. Schmitt).

One Symposium on Chrysomelidae, the First European (but perhaps the 7a), was held in Hungary (Fig. 14), on Buda side of Budapest and organized by Karoly Vig and Michael Schmitt. A beautiful evening boat trip, with an excellent dinner and adapted music, along the blue Danube, closed the 9th European Congress (August 22 to 27, 2010). Here, Eduard Petitpierre talked about chromosomal evolution. Many very interesting papers were presented including one by the Japanese rising star Yoko Matsumura (Fig. 15).

**Figure 16.**
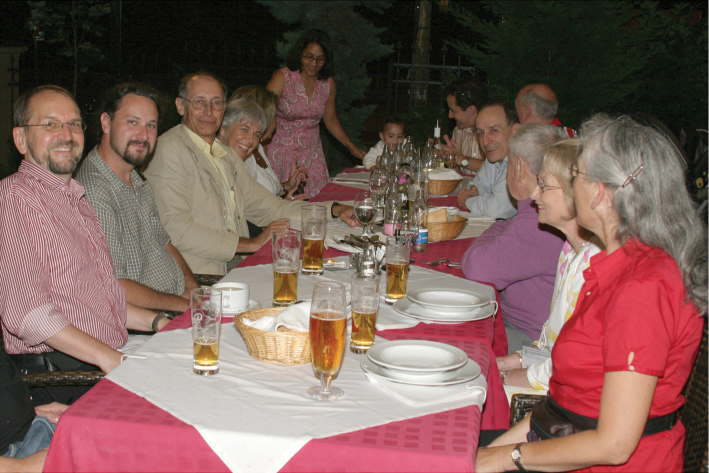
Budapest (Hungary), 23 August, 2010, joint dinner (from left: Michael Schmitt, Gabor Pszodai, Eduard Petitpierre, Carmelèn Petitpierre, Caroline Chaboo’s mother-in-law, Caroline Chaboo, her daughter Teresa and her husband Fernando, (Caroline’s father-in-law), Carlo Leonardi, Pierre Jolivet, Madeleine Jolivet, Elisabeth Geiser (photo: M. Schmitt).

Two Turkish colleagues (Ali Gök and Ismail Sen) were present, and at the European dinner, in an inn nearby, Mauro Daccordi and Carlo Leonardi appeared coming from nowhere (Fig. 16).

Caroline Chaboo with her family was there also. She gave a very interesting tortoise beetle talk. Michael Schmitt succeeded to have the virtual 7th symposium published through L. Penev in Research on Chrysomelidae, volume 3, by Pensoft, Sofia.

**Figure 17.**
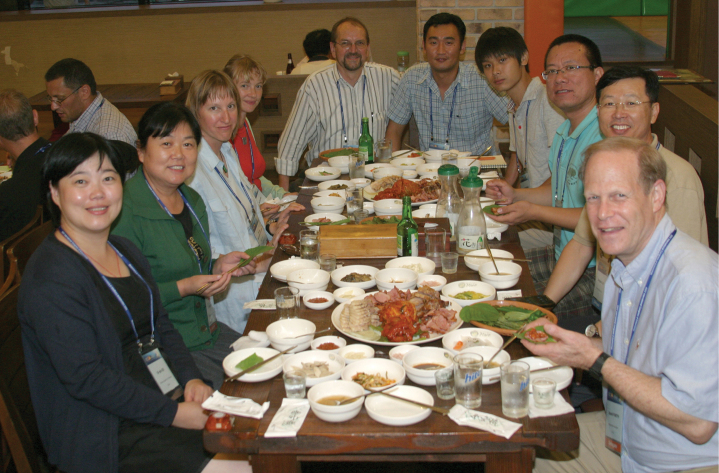
A Korean dinner, after the 8th International Symposium on the Chrysomelidae, Daegu (South Korea), 23 August, 2012 (from left): Si Qin Ge, Jun-zhi Cui, Nicole Kalberer-Simmen, Antje Burse, Michael Schmitt, Choru Shin, Haruki Suenaga, Mai Bing, Jong Eun Lee, David Furth (photo: M. Schmitt).

The official Eighth International Symposium on the Chrysomelidae was held in Daegu (Fig. 17), South Korea, on 23^rd^ of August, 2012, in conjunction with the 24th International Congress of Entomology. That was the first Congress that I missed, and I hesitated very much, pain in my knees were responsible for my absence. I have worked in Korea during 3 years before and I knew the place, where I once collected beetles and organized aerial sprays. We used at that time the US base as a hotel, but that was many years ago. The proceedings of this meeting were printed within volume 4 of Research on Chrysomelidae, within ZooKeys, Pensoft, a normally electronic publication but which can be printed into a book. This 8th Symposium was organized by Michael Schmitt and Jong Eun Lee. Many first class papers were presented including one with the Chinese rising star of Chrysomelidae Si Qin Ge. David Furth was there with the Mexican Alticinae, Donald Windsor, Michael Schmitt, and some others presented well documented papers on various topics. The text of all those communications was available free of charge, but printing of this virtual book remains quite expensive. A success, this symposium, which precedes two more in the future, one in York, UK, in August 2014, perhaps also virtual, with the 10th European Congress of Entomology, and the 25^th^ International Congress of Entomology, in September 2016, in Orlando, Florida, coordinated with the Entomological Society of America and a few other groups.

The Chrysomelidae International Symposia were always held in connection with the International Congresses of Entomology every 4 years and in between with the European Congresses of Entomology, also every four years, with few exceptions. Generally, the organizer was David Furth with a local chrysomelidologist. Actually Michael Schmitt, for the last four Chrysomelidae Symposia, took over the organization of those meetings.

## Progress in Chrysomelidology

Numerous were the discoveries made during those last 30 years. Let us quote some of them: metafemoral spring of flea beetles and jumping by David Furth (1988, Furth and Suzuki 1994, 1998), and Michael Schmitt (2004); meioformulae of Leaf Beetles by Petitpierre (1997, 1999, 2011), Virkki (1985, 1988, 1989) and others; larvae research, by Steinhausen (1985, 1994, 1995, 1996) and others; chemical defense by Jacques Pasteels (Pasteels and Hartmann 2004, Pasteels and Rowell-Rahier 1989, Pasteels et al. 1986, 1988, 1989, 1994, 1996, 2004); cycloalexy, by Vasconcellos-Neto and Jolivet (1989, 1994); fossils by Santiago-Blay (1994; Santiago-Blay and Craig 1999, Santiago-Blay et al. 1996, 2004), followed by many others; mimicry by Balsbaugh (1988, Balsbaugh and Fauske 1991) and many others; zoogeography by Verma (Verma and Jolivet 2004, 2006), Scherer (1988), Daccordi (1994, 1996, 2000, 2003a, b, c), and many more; egg bursters by Cox (1988, 1994); structure of ovaries and viviparity by Christian Bontems (1988, Bontemps and Lee 2008); Criocerinae biology, by Fredric Vencl (Vencl and Morton 1998, 1999, Vencl and Nishida 2008, Vencl et al. 2004), M. Schmitt (1988), Yoko Matsumura (Matsumura and Akimoto 2009, Matsumura and Suzuki 2008, Matsumura and Yoshizawa 2010, 2012, Matsumura et al. 2010, 2012); African fauna of Alticinae by Maurizio Biondi (1989, 1999, 2001a, b, Biondi and D’Alessandro 2008, 2010a, b, 2012); Australian fauna by Mauro Daccordi (2000, 2003a, b, c, Daccordi and DeLittle 2003), Chris Reid (1989, 1991a, b, 1992, 2003, 2006, Reid and Beatson 2010a, b); colour and changes of colour by Jean-Pol Vigneron (Vigneron et al. 2007); biology of Tortoise Beetles by Fredric Vencl (Vencl and Allen 2006, Vencl and Srygley 2013, Vencl et al. 2004, 2013), Caroline Chaboo (2001, 2002, 2004, 2007, Chaboo and Nguyen 2004, Chaboo et al. 2014), Don Windsor (1987, Windsor and Choe 1994, Windsor et al. 2013) etc.; Chinese and Far East fauna, by Shi-xiang Chen (1985, Chen and Zia 1984a, b, Chen et al. 1985), Shinsaku Kimoto (1984, 1988, 2005), Mohamed Mohamedsaid (1990, 1991, 1992, 1993a, b, 1994, 1995, 1999, 2004, 2009, Mohamedsaid and Constant 2007, Mohamedsaid and Takizawa 2008), Haruo Takizawa (2007); Taiwan and Japanese fauna by Shinsaku Kimoto, Haruo Takizawa (Kimoto and Takizawa 1997), Jong Eun Lee (1991, 1993, Cho and Lee 2005, Park et al. 2012), and others; biology and taxonomy of Aulacoscelidinae by Don Windsor (Windsor et al. 1999), Jorge Santiago-Blay (2004), and others and its behaviour on cycads (*Zamia*); biology of *Oreina* by Martine Rahier (Rowell-Rahier and Pasteels 1994), and so many other papers. Larvae of Aulacoscelidinae are known, but, as for the Orsodacninae, we still are not sure where the larvae develop and on which plant. Attraction by cycads does not seem to be only pharmacology. We do not know anything about the development of the larvae of Australian Sagrinae, some with free pupae, others with pupation inside the stem. Pupation inside the stem seems sometimes the rule for Spilopyrinae (in New Caledonia at least). DNA barcodes were used to recognize the host-plants eaten by leaf beetles (Australian Chrysomelinae) from their gut contents and showing their evolutionary implications for insect-host plants interactions (Jurado-Rivera et al. 2009).

Terry Seeno and John A. Wilcox contributed to the clarification of the classification in 1982, as well as later on Kunio Suzuki (1996). Since then many new species and genera were described, mostly among Eumolpinae, Chrysomelinae, Alticinae, Galerucinae, and others. Host-plants of the group tend to be known more and more, and practically it has been deciphered for most of the Holarctic. In the tropics, we have many references, but still a lot of observations are requested. Too many missing data remain in Brazil, Africa and Australia (as for Sagrinae). Also many species, chiefly among Eumolpinae, can be rather polyphagous, when Chrysomelinae are mostly stenophagous. There are even in the tropics extremely rare cases of carnivory among *Diabrotica* (Mafra-Neto and Jolivet 1994). Clytrinae, some Eumolpinae and Cryptocephalinae can be myrmecophilous in East Africa within *Acacia* domatia. It is a field, myrmecophily, where research should be deepened, mainly in the tropics. Surprises can be expected. We know very little about Neotropical Clytrinae biology. According to William Eberhard (1996), female Chrysomelidae probably show also a cryptic selection of the male.

In South Africa, several beetles copy superficially the *Timarcha*. Beth Grobbelaar is going to clarify all the *Iscadida* mysteries (egg laying, food-plants, distribution). A very peculiar biology for a false timarchoid adapted to dryness and to a Mediterranean-type climate. More should be discovered about the biology of the South African timarchoids.

Problems arise also on the holes on elytra of certain leaf beetles sometimes in connection with glands or sensitive detection cells.

## Books on Chrysomelidae

**Figure 18.**
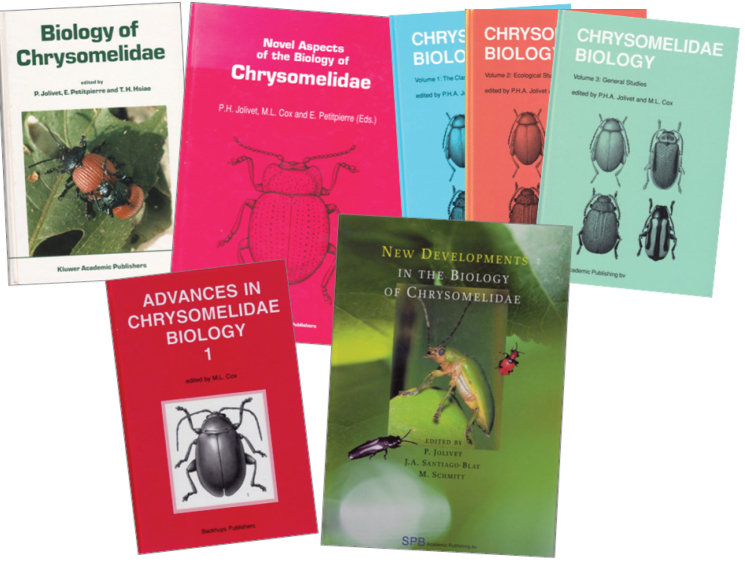
"The Books" on Chrysomelidae, except the proceedings volumes of the International Symposia: Jolivet P, Petitpierre E, Hsiao TH (Eds.) 1988, Jolivet PH, Cox ML, Petitpierre E (Eds) 1994, Jolivet JHA, Cox ML (Eds.) 1996 (3 vols.), Cox ML (Ed) 1999, Jolivet P, Santiago-Blay JA, Schmitt M (Eds.) 2004.

Outside the publications of the International symposia on Leaf Beetles, books were regularly published on the topic in the Netherlands, France, Germany, Russia, Poland, USA, sometimes in correlation, sometimes independently of the symposia (Fig. 18).

There are also the "festschrift" books (e.g., Borowiec and Furth 2007, Konstantinov et al. 2005, Pakaluk and Slipinski 1995), the monographs, etc. There are two "sacred" species groups among the chrysomelids: *Leptinotarsa decemlineata*, the Colorado Potato Beetle, and *Timarcha* spp., the bloody nose beetles, on which there are hundreds of publications. I am not including here the books or booklets on the Colorado Potato Beetle, the worshippers being, in the past, separated during the Congresses from the "real" chrysomelidologists, as for Ferro and Voss booklet (1985). Bruchidologists have traditionally, sadly, met the same fate. In both cases, it is more agricultural entomology. *Diabrotica virgifera*, sadly famous in the US, has actually invaded Europe and becomes also the subject of many books and articles.

## Conclusions

Many discoveries were made the last 30 years in the field of Chrysomelidology. Since Chapuis, Jacoby and others in the past, and more recently since Chen (1964), there has been some evolution in the placement of subfamilies. The passing of Chen, Crowson, Bechyné, Chûjô, Balsbaugh, Edwards, Wilcox, Iablokoff, Lopatin, Ruffo, Kimoto, Kaszab, Erber, Monrós, Scherer, Verma, Yu Peiyu, LeSage and so many others, were a great loss for chrysomelid taxonomy and biology. New stars appear, either in taxonomy or in biology, using new techniques. Some just pass as quick as a flash. Others remain faithful to the topic and the relief is ready. We owe to Roy Crowson many discoveries on the Chrysomelidae, including the perception of Spilopyrinae and of the peculiar *Eupales*, the study of Sagrinae, and the researches on some Galerucinae, and others INBio begun by Dan Janzen in La Selva, Costa Rica and STRI in Panama (Windsor, Flowers, Vencl, etc.) were also important centres of research on leaf beetles.

Some changes in taxonomy have also been proposed, based on simple morphology. Cladistics and molecular biology inspired some others (Hsiao, Farrell, Duckett, Gomez-Zurita, Reid and many others). On some big changes, I do not fully agree, mostly on the breaking of the family Chrysomelidae and the merging of Alticinae and Galerucinae, of Cassidinae and Hispinae. They are intermediary taxa. One subfamily has really merged with Eumolpinae: Megascelidinae (Jolivet 1957-1959) and one is probably correctly separated from Eumolpinae, the Spilopyrinae (Reid 2000). Those are, however, primitive Eumolpinae, but with different genitalia and behaviour. It was a feeling of Crowson and confirmed by Reid. Synetinae are an aberrant group, well characterized, and that makes for Chrysomelidae 19 subfamilies, at least for me. Recent new classifications separated Chrysomelidae and other supposedly closely related families(?) (Orsodacnidae and Megalopodidae). There are splitters for families as they exist also for species. Chrysomelidae are related also to Bruchidae, often now classified into Chrysomelidae, despite the opposition of some famous bruchidologists, as John Kingsolver (1995), Krishna K. Verma (Verma and Saxena 1996), etc. Here, splitters become mergers, but this is a personal decision, a free act. However, in agricultural journals, we find Bruchidae or Bruchinae according to the secret feelings of the authors of the papers and their convictions. *Rhaebus* and *Eubaptus* are transitional between bruchids and sagrines, as there exist also transitional genera between Cassidinae and Hispinae and between Alticinae and Galerucinae. Synetinae however seem to remain completely isolated.

I am very sorry if I forget some of our chrysomelidologist friends and their publications. This is not intentional. There were many in the past and a lot during those last 30 years, from many countries and continents. I am not sure to have them all in my list. Please forgive me, many being faunas and not in direct connection with the symposia. Sometimes those local faunas are in the language of the country.

It is certain that some areas need more investigation, as Madagascar for instance, and that there remain many biological problems to be solved or to be discovered. The fauna is near to be well investigated in Europe, in the US, in Japan, Australia and in China. Still Indonesia, tropical America, India, Malaysia, Vietnam, tropical Africa, New Guinea can bring us some novelties, but deforestation reduces the number of species and genera, and many will disappear before being described. Few will persist as fossils in the tropics. Millions of Insects have existed in the past and will remain unknown forever.

Orsodacninae are distributed all along the Holarctic area and Aulacoscelidinae are restricted to the Neotropics. We do not know for sure where and on which plants the larvae develop. Archaic Australian Sagrinae are also practically unstudied regarding biology and development. They come to light, but are rarely discovered near a possible host plant. Eight symposia on Chrysomelidae have been held. Many new things have been found but some problems remain unsolved.

De Gruyter treatise of Zoology (Leschen and Beutel 2014) has put up to date the classification of the group. No doubt that our successors refine the system and perhaps will come back to a more reasonable grouping.
